# Evaluation of potential mechanisms for skeletal muscle mass recovery early after left ventricular assist device implantation

**DOI:** 10.1016/j.jhlto.2025.100338

**Published:** 2025-07-04

**Authors:** Didjana Celkupa, Benjamin A. Sweigart, Joronia Chery, Alex Coston, Laura Telfer, Matthew Lawrence, Michael S. Kiernan, Gregory S. Couper, Masashi Kawabori, Nathan LeBrasseur, Edward Saltzman, Amanda R. Vest

**Affiliations:** a CardioVascular Center, Tufts Medical Center, Boston, MA; b Biostatistics, Epidemiology, and Research Design (BERD) Center, Tufts Clinical and Translational Science Institute, Tufts Medical Center, Boston, MA; c Tufts University School of Medicine, Boston, MA; d Department of Family and Community Medicine, San Francisco General Hospital, University of California San Francisco, San Francisco, CA; e Penn State College of Medicine, State College, PA; f Department of Physical Medicine and Rehabilitation, and Robert and Arlene Kogod Center on Aging, Mayo Clinic, Rochester, MN; g Friedman School of Nutrition Science and Policy at Tufts University, Boston, MA; h Kaufman Center for Heart Failure Treatment and Recovery, Department of Cardiovascular Medicine, Heart, Vascular and Thoracic Institute, Cleveland Clinic, Cleveland, OH

**Keywords:** Heart failure, Skeletal muscle, Lean mass, Metabolism, Physical activity, Nutrition, Left ventricular assist device

## Abstract

**Background:**

We observed significant gains in appendicular lean mass (ALM) over the first 6 months of left ventricular assist device (LVAD) support for patients with heart failure with reduced ejection fraction (HFrEF). We sought to determine whether improved HF neurohumoral stability, inflammation, physical activity, or nutrition, are most closely related to this muscle mass recovery.

**Methods:**

We prospectively recruited 30 adults with HFrEF ±21 days from LVAD implantation. Dual X-ray absorptiometry (DXA) measured ALM at baseline and at 3- and 6-months post-LVAD implantation (*n* = 22 with ALM at baseline and 3 months). Markers of neurohumoral HF stability (NT-proBNP, growth differentiation factor-15), inflammation [high sensitivity C-reactive protein (hsCRP)], habitual physical activity (24-hour average steps), and nutritional intake (24-hour average dietary protein) were also recorded. Mixed effects models separately evaluated the change in each parameter over time and relationships with the change in ALM.

**Results:**

At baseline, participants (87% male, mean age 56 ± 12 years) showed a significant negative association between ALM and log N-terminal-pro B natriuretic peptide (NT-proBNP) (*r* = −0.38, 95% CI −0.66, −0.001, *p* = 0.050) and log growth differentiation factor-15 (GDF-15) (*r* = −0.42, 95% CI −0.69, −0.05, *p* = 0.027). Over the 6-month study period, NT-proBNP and hsCRP decreased, 24-hour steps increased, whereas GDF-15 and 24-hour dietary protein were unchanged. There was an increase in ALM across study timepoints, which was significantly associated only with reductions in log NT-proBNP and hsCRP on mixed effects models.

**Conclusions:**

The recovery in ALM over the first 6 months of LVAD support was most closely associated with improved HF neurohumoral stability and inflammation, rather than activity or nutritional changes.

## Background

Skeletal muscle wasting is a common occurrence among patients with heart failure (HF), observed in at least 10%-20% of ambulatory patients with HF and up to approximately 50% of patients with advanced HF with reduced ejection fraction (HFrEF).^1^, ^2^, ^3^, ^4^, ^5^ Muscle wasting and unintentional weight loss are associated with increased mortality among patients with HF,^6^, ^7^, ^8^, ^9^, ^10^ with an adjusted hazard ratio of 2.63 (95% confidence interval (CI) 1.35-5.12, *P* = 0.005) in one seminal ambulatory HFrEF cohort.^6^ For patients with HF who are experiencing muscle wasting, there are currently no approved pharmacotherapies to help regain lost weight or muscle mass.^11^ Additionally, the clinical drivers of muscle loss are undefined. Potential contributors to wasting include: 1) neurohumoral and inflammatory activation in decompensated HF driving a catabolic metabolic state; 2) decreased physical activity leading to muscle atrophy; and 3) insufficient dietary caloric intake to support muscle maintenance.

In current clinical practice, patients with HF and catabolic weight loss are often counseled to increase dietary protein-calorie intake. However, it is unknown whether increasing caloric intake, for example through oral protein supplementation or tube feeding, is effective in preventing or reversing muscle wasting in the setting of a persistently activated neurohumoral state during the advanced HF syndrome.^11^ One scenario in which muscle wasting can be reversed is after stabilization of the HF syndrome with left ventricular assist device (LVAD) implantation, where patients commonly gain weight, especially if underweight at baseline.^12^, ^13^ We observed statistically and clinically significant gains in total body mass and lean mass over the first 6 months of LVAD support, with a mean fat free mass (FFM) change at 3 and 6 months after LVAD implantation of 2.3 kg (95% CI 1.0, 3.5) and 4.2 kg (95% CI 2.2, 6.1), respectively.^1^ The minor increase in fat mass in this cohort was not statistically significant. In another LVAD cohort, gains in body mass index (BMI) occurred after improved HF neurohumoral stability as represented by a reduction in N-terminal-pro B natriuretic peptide (NT-proBNP); furthermore, a reduction in NT-proBNP was necessary for recovery of BMI to occur.^14^

Understanding potential mechanisms of muscle mass recovery for LVAD recipients may guide the development of new pharmacological, physical activity, or nutritional interventions that reverse skeletal muscle wasting for the wider population of patients with HFrEF and catabolic weight loss who will not receive an LVAD. Therefore, the primary objective of this observational analysis was to determine which of the following candidate mechanisms shows the strongest relationship with the recovery of lean mass previously observed by dual X-ray absorptiometry (DXA) imaging in our cohort of 30 LVAD recipients: 1) improved HF neurohumoral stability and inflammation; 2) increased habitual physical activity; and 3) improved dietary protein-calorie intake. We hypothesized that recovery of lean mass would be more closely related to HF neurohormonal and inflammatory recovery than with increases in physical activity or protein-calorie intake. This was based upon prior observations of the recovery in multiple metabolic parameters at 3-6 months of LVAD support, including total cholesterol, hemoglobin A1c, NT-proBNP, growth differentiation factor (GDF)-15, and C-reactive protein (CRP), which temporally correlate with the prognostically important weight gain observed in the Intermacs LVAD cohort.^10^, ^14^, ^15^, ^16^, ^17^ An additional objective was to characterize the relationship between improvements in muscle mass and changes in muscle strength, with the hypothesis that changes in muscle mass and strength would be closely associated.

## Methods

Participants were prospectively recruited from all adults with advanced HFrEF undergoing evaluation for LVAD implantation at Tufts Medical Center between August 2017 and December 2019. Study procedures were performed within 21 days before or after LVAD implantation, with the rationale for study design and details of these methods published previously.^1^ Study visit activities were repeated at 3 and 6 months after LVAD implantation (each ±14 days), with study participation concluding either after the 6-month visit or at the time of heart transplantation, whichever occurred first. Study participants were managed as per institutional standard of care, including routine pre-LVAD nutritional counseling, echocardiographic-guided LVAD speed optimization prior to hospital discharge, reintroduction of HFrEF medical therapies as indicated, and referral to outpatient cardiac rehabilitation around 3 months post-LVAD implantation. This observational study was approved by the Tufts Health Sciences Institutional Review Board and written informed consent was obtained from all subjects before participation.

### Baseline characteristics

Participants’ age, sex, duration of HF, HF etiology, major comorbidities, pre-LVAD temporary mechanical support, LVAD strategy, Intermacs class, and concominant heart faulire medications were recorded from the medical chart. BMI was calculated as weight (kg) measured by standing scales (excluding the weight of the LVAD controller, if present) divided by height (m)-squared.

### Skeletal muscle mass assessment

Participants underwent whole body DXA (Hologic, Marlborough, MA; Discovery A model or Hologic Horizon A model) in the supine position with the external LVAD controller and batteries positioned outside the DXA scan field. All scans were analyzed on the Hologic Horizon A machine with Whole Body Analysis software v.5.6.05 and the National Health and Nutrition Examination Survey calibration function “National Health and Nutrition Examination Survey BCA” enabled. The primary metric for skeletal muscle mass in this analysis was the appendicular lean mass (ALM), which is the lean mass in the upper and lower extremities without bone mineral content. ALM was selected in preference to FFM due to the theoretical possibility that FFM could be positively biased by the implantation of the LVAD pump in the thoracic region and because FFM includes organ mass in addition to skeletal muscles. Due to the logistical challenges of obtaining the baseline DXA scan near the LVAD implantation date as previously described,^1^ there was a mean 30-day interval between the blood, activity, and nutrition testing and the baseline DXA scan.

### HF neurohumoral stability and inflammation assessment

Venous blood was drawn during study visits in the fasted state. For the biomarker's measurements, blood was processed and stored in a −80 freezer with no freeze-thaw cycles. Serum samples were analyzed in bulk for NT-proBNP as a biomarker of HF stability, using electrochemiluminescence. The primary metric of HF neurohumoral stability was NT-proBNP, given its established role as a biomarker of HF diagnosis and prognosis.^18^ The plasma concentration of GDF-15 was quantified using a commercially available magnetic bead-based immunoassay (R&D Systems, Minneapolis, Minnesota) on the Luminex xMAP multianalyte profiling platform and analyzed on a MAGPIX System (Merck Millipore, Burlington, Massachusetts). GDF-15 was selected because it is both a biomarker of HF stability,^19^, ^20^, ^21^ and is thought to have a direct catabolic effect that promotes anorexia and muscle wasting in other disease states.^22^, ^23^ Systemic inflammation was assessed by high sensitivity C-reactive protein (hsCRP), using a particle enhanced immunoturbidimetric assay. hsCRP was run on venous blood that was not stored prior to assay.

### Habitual physical activity assessment

Subjects wore an ActiGraph GT9X-BT device (ActiGraph LLC, Pensacola, Florida) on the wrist for an intended 7-day period (minimum 3 days) after study visits. The ActiLife version 6.13.4 software was used to calculate 24-hour average steps and the percent active time. Given that nearly all recorded activity was classified as light, with minimal engagement in moderate or vigorous activity, separately reporting percent active time spent in these categories was deemed low-yield. The primary metric of physical activity was 24-hour average steps due to its feasibility and reliability in this patient population.

### Dietary intake assessment

Subjects completed 3 facilitated non-consecutive 24-hour food recalls around each study visit time point, reporting all meals, snacks, and beverages consumed during the 24-hour periods. Data were entered into the Food Processor database (ESHA Research, Salem OR) for quantification of macronutrient and micronutrient intake, with each nutritional component expressed as a 24-hour average across the 3 days of food recall. Protein intake was indexed to bodyweight as grams (g) per kilogram (kg) of bodyweight per day, and the primary metric of dietary adequacy was the 3-day average 24-hour dietary protein in g/kg. Averaged daily grams of carbohydrate and fat intake were also tabulated, and additionally expressed as the averaged percentage of total caloric intake by multiplying carbohydrates by a factor of 4 and fats by a factor of 9 to convert from grams to kcal. Each of these nutritional parameters was expressed as mean change from baseline to 3 or 6 months, with a 95% CI. Participants also completed the Short Nutritional Assessment Questionnaire, where scores ≥3 indicate a high risk of malnutrition.^24^, ^25^

### Muscle strength, physical function, and patient reported health status assessment

Each study visit also included an assessment of exploratory variables representing skeletal muscle strength and physical function. Handgrip strength was measured for 10 sec with the dominant hand, averaging 3 attempts with a hydraulic hand dynamometer (Baseline Evaluation Instruments Fabrication Enterprises Inc., Irvington, NY). The short physical performance battery (SPPB) is a validated test assessing balance, gait, mobility, and endurance. It measures the subject’s ability to stand for up to 10 sec with feet in the side-by-side, semi-tandem, and tandem positions, the time to complete a 4-m walk and the time to rise from a chair and return to the seated position 5 times.^26^, ^27^, ^28^ SPPB is scored out of 12, and scores ≤8 represent impaired physical function. A further assessment of physical function was performed using the 6-min walk test (6MWT). Patient reported health status was assessed using the 23-item Kansas City Cardiomyopathy Questionnaire (KCCQ, CV Outcomes Inc, Kansas City, MO),^29^, ^30^ a validated self-administered HF health status measurement tool that quantifies physical limitations, symptoms, self-efficacy, social interference, and quality of life. The summary score ranges from 0 to 100, with higher scores indicating better health status.

### Statistical methods

Continuous variables were expressed as means ± standard deviations or medians with 25th and 75th percentiles, depending upon data normality. Categorical variables were tabulated as values and percentages. NT-proBNP and GDF-15 values were log-transformed prior to statistical analysis due to their skewed distributions. The Pearson correlation statistics were calculated for the relationship between the baseline ALM and the baseline NT-proBNP, log GDF-15, and hsCRP, as these variables were all normally distributed. Mean changes from baseline to 3 and 6 months were calculated with 95% CI for key body composition, HF stability, physical activity, and nutrition parameters. Changes over time were displayed graphically using box-and-whisker plots representing the median as a line, the 25th and 75th percentiles as the box boundaries, and the minimum to maximum range of data as whiskers.

We then evaluated changes across the three study visits in ALM, log-transformed NT-proBNP, log-transformed GDF-15, hsCRP, 24-hour average steps, and 24-hour averaged protein intake with linear mixed effects models. These models included time as a categorical variable and a random intercept for each subject. We also evaluated the relationship between DXA ALM as an outcome and the biomarker, activity, and nutritional parameters (log-transformed NT-proBNP, log-transformed GDF-15, hsCRP, 24-hour average steps, and 24-hour averaged protein intake, all subject-mean centered to separate with- and between-participant effects) using additional linear mixed effects models. All the models included time as a categorical variable. We fit additional models adjusted for age, sex, and HF duration. Similar models were generated for the exploratory secondary assessment metrics of handgrip strength, SPPB, 6MWT, and KCCQ summary score. Mixed effects models were generated using SAS version 8.3 update 3 (SAS Institute Inc., Cary, NC) and *p*-values < 0.05 was considered statistically significant.

To complement the mixed effects models that evaluated relationships in changes in absolute values over the 6-month period, we also used a percent change approach across all candidate variables that included visualization of individual participant data to facilitate detection of outlier values.

The percent changes from baseline to 3 months and baseline to 6 months were calculated for ALM and the biomarker, physical activity, and protein intake metrics and visually displayed on scatterplots. Spearman correlation coefficients between percent change in ALM and each of these metrics were calculated, given the non-normal distribution of several variables. Prism 9.1.2 for MacOS was used for descriptive statistics, correlation calculations, and figures (GraphPad Software, Inc., San Diego, CA).

## Results

### Baseline characteristics

Thirty LVAD recipients contributed data to this study, as previously reported.^1^ The cohort was predominantly male (87%), with baseline mean age 56 ± 12 years (age range 31-74 years), 50% HeartWare LVAD and 50% HeartMate 3 LVAD implants, and a bridge-to-transplantation LVAD strategy for 70% of participants (Supplemental Table 1a). At baseline, 70% of patients were on standing loop diuretics, 20% on beta blockers, and 30% on angiotensin converting enzyme inhibitors, angiotensin receptor blockers, or angiotensin receptor neprilysin inhibitors. By 3 months, medication use shifted to 64%, 36%, and 25%, respectively, and by 6 months to 60%, 35%, and 35% (Supplemental Table 1b). There were no mortalities over the 6-month period of the study, but 67% of patients had a hospital readmission within the first 3 months of the study, and 100% had a readmission by 6 months.

### Skeletal muscle mass

**Figure 1:**
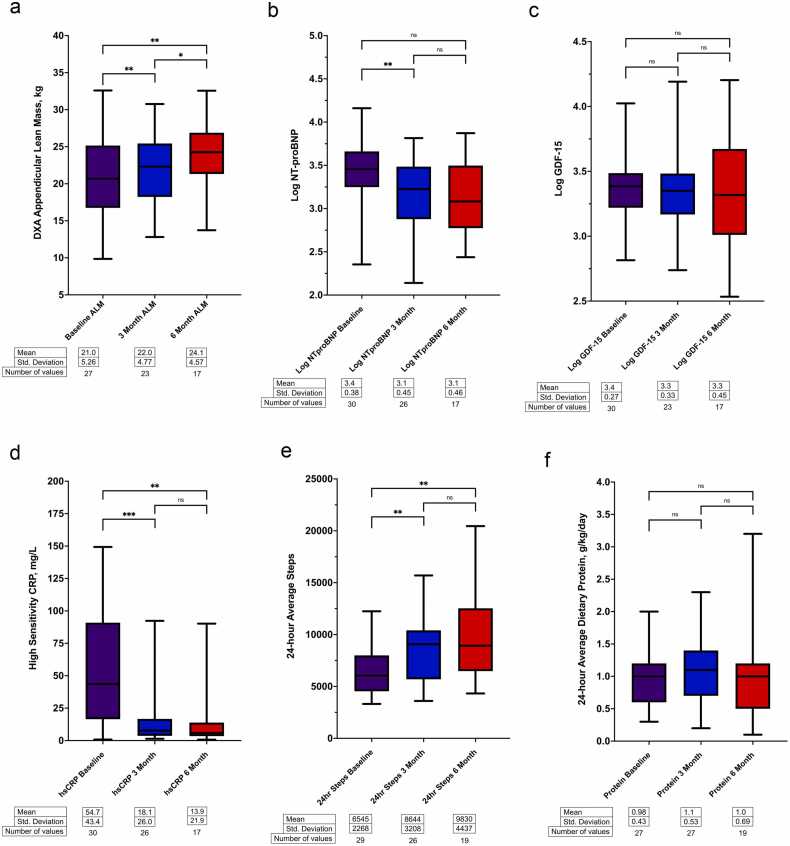
*Box plots of change over time.* Paired *T* testing between time points: * *p* ≤ 0.05; ** *p* ≤ 0.01; *** *p* ≤ 0.001; ns non-significant. *Abbreviations:* ALM, appendicular lean mass; DXA, dual X-ray absorptiometry; GDF-15, growth differentiation factor 15; hsCRP, high sensitivity C-reactive Protein; kg, kilogram; LVAD, left ventricular assist device; NT-proBNP, N-terminal pro B natriuretic peptide. **(a)** Change in Appendicular Lean Mass Over Time, **(b)** Change in Log NT-proBNP Over Time. **(c)** Change in Log GDF-15 Over Time. **(d)** Change in High Sensitivity C-Reactive Protein Over Time. **(e)** Change in 24-hour Average Steps Over Time. **(f)** Change in 24-hour Average Dietary Protein Intake Over Time. (Reproduced with permission from Vest AR, Wong WW, Chery J, Coston A, Telfer L, Lawrence M, Celkupa D, Kiernan MS, Couper G, Kawabori M and Saltzman E. Skeletal Muscle Mass Recovery Early After Left Ventricular Assist Device Implantation in Patients With Advanced Systolic Heart Failure. *Circ Heart Fail*. 2022:101161CIRCHEARTFAILURE121009012.)

As previously reported, there was a significant increase in ALM during the first 6 months of LVAD support: baseline 21.0 ± 5.3 kg; 3 months 22.0 ± 4.8 kg; 6 months 24.1 ± 4.6 kg; mixed-effects joint test *p* < 0.0001; Table 1; Figure 1a).^1^ The change over time in ALM remained significant after adjustment for age, sex, and HF duration (adjusted mixed-effects joint test *p* < 0.0001, Table 2).

**Table 1:** Changes in Body Composition, Biomarker, Physical Function and Activity, and Dietary Intake Parameters During LVAD Support

DXA body composition	Baseline (*n* = 27)	3 months (*n* = 23) *Mean Change from Baseline (95% CI)*	6 months (*n* = 17) *Mean Change from Baseline (95% CI)*

Appendicular lean mass, kg	21.0 ± 5.3	22.0 ± 4.8 *1.5 (0.7, 2.3)*	24.1 ± 4.6 2.3 (0.9, 3.6)
Fat free mass, kg	56.4 ± 11.7	57.7 ± 11.2 *2.3 (1.0, 3.5)*	62.3 ± 10.9 *4.2 (2.2, 6.1)*
Total mass, kg	80.8 ± 19.4	82.8 ± 17.9 *2.4 (0.3, 4.6)*	90.0 ± 16.9 *5.5 (1.4, 9.6)*

Markers of HF neurohumoral stability and inflammation	Baseline (*n* = 30)	3 months (*n* = 26 NT-proBNP, *n* = 23 GDF-15, *n* = 26 hsCRP) *Mean Change from Baseline (95% CI)*	6 months (*n* = 17 NT-proBNP, GDF-15 and hsCRP) *Mean Change from Baseline (95% CI)*

NT-proBNP	3728 ± 3308	2107 ± 1756 *−1849 (−3247, −451)*	2197 ± 2374 *−1309 (−3278 to 661)*
GDF-15	2926 ± 2144	2904 ± 3071 *−383 (−1310, 544)*	3342 ± 3902 *196 (−1109, 1501)*
High-sensitivity CRP	54.69 ± 43.41	18.14 ± 26.01 *−36.11* *(−54.97, −17.25)*	13.86 ± 21.88 *−38.73* *(−61.83, −15.63)*

CI, confidence interval; CRP, C-reactive protein; DXA, dual X-ray absorptiometry; GDF-15, growth differentiation factor 15; kg, kilogram; LVAD, left ventricular assist device; NT-proBNP, N-terminal pro B natriuretic peptide. Continuous variables expressed as means ± standard deviations. Changes from baseline expressed as mean change and (95% CI). Notes: One participant with DXA images at the 3- and 6-month visits had no baseline DXA for paired comparisons.

**Table 2:** Summary of Linear Mixed Effect Models for Changes in Appendicular Lean Mass, Biomarker, Physical Activity, and Dietary Intake Parameters Over Time

Outcome variable	Adjusted*	3-month change (95% CI)	*P* value	6-month change (95% CI)	*P* value	*P* value joint test
DXA ALM	No	1.50 (0.66, 2.34)	0.0009	2.32 (1.37, 3.26)	<0.0001	<0.001
DXA ALM	Yes	1.54 (0.70, 2.38)	0.0007	2.34 (1.39, 3.28)	<0.0001	<0.001
Log NT-proBNP	No	−0.29 (−0.47, −0.10)	0.003	−0.28 (−0.49, −0.06)	0.012	0.005
Log NT-proBNP	Yes	−0.29 (−0.48, −0.11)	0.003	−0.28 (−0.50, −0.07)	0.011	0.004
Log GDF-15	No	−0.19 (−0.44, 0.07)	0.15	−0.19 (−0.48, 0.10)	0.18	0.250
Log GDF-15	Yes	−0.19 (−0.46, 0.06)	0.12	−0.20 (−0.49, 0.09)	0.18	0.221
hsCRP	No	−38.1 (−54.7, −21.4)	<0.0001	−41.1 (−60.4, −21.7)	0.0001	<0.001
hsCRP	Yes	−37.1 (−53.7, −20.5)	<0.0001	−40.8 (−60.1, −21.6)	0.0001	<0.001
24 hours Steps	No	2291 (734, 3848)	0.005	3499 (1762, 5236)	<0.001	<0.001
24 hours Steps	Yes	2313 (752, 3875)	0.005	3476 (1723, 5230)	<0.001	<0.001
24 hours Protein intake	No	0.09 (−0.15, 0.34)	0.43	0.09 (−0.18, 0.36)	0.51	0.69
24 hours Protein intake	Yes	0.09 (−0.15, 0.33)	0.46	0.09 (−0.18, 0.36)	0.52	0.71

* Adjusted for age, sex, and HF duration.

### HF neurohumoral stability and inflammation

**Figure 2:**
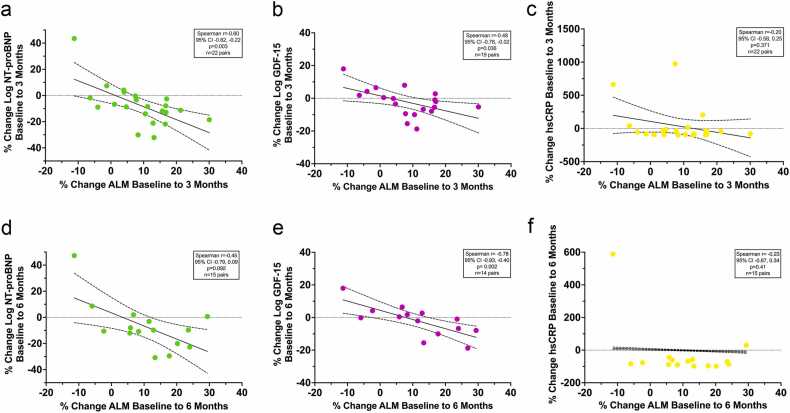
Percent change in appendicular lean mass and biomarkers. *Abbreviations:* ALM, appendicular lean mass; DXA, dual X-ray absorptiometry; GDF-15, growth differentiation factor 15; hsCRP, high sensitivity C-reactive Protein; NT-proBNP, N-terminal pro B natriuretic peptide. **(a)** Relationship between change in Log NT-proBNP and change in appendicular lean mass over 3 months. **(b)** Relationship between change in Log GDF-15 and change in appendicular lean mass over 3 months. **(c)** Relationship between change in hsCRP and change in appendicular lean mass over 3 months. **(d)** Relationship between change in Log NT-proBNP and change in appendicular lean mass over 6 months. **(e)** Relationship between change in Log GDF-15 and change in appendicular lean mass over 6 months. **(f)** Relationship between change in hsCRP and change in appendicular lean mass over 6 months. (Subject 10 was observed to be an outlier in the relationship between both NT-proBNP or GDF-15 and ALM, but notably this was the only participant to develop advanced renal failure during the LVAD follow-up period (subject 10 was retained within the analysis).)

Baseline ALM was negatively correlated with baseline log NT-proBNP (*r* = −0.38, 95% CI −0.66, −0.001, *p* = 0.050) and log GDF-15 (*r* = −0.42, 95% CI −0.69, −0.05, *p* = 0.027), but not the hsCRP (*r* = −0.12, 95% CI −0.48, 0.27, *p* = 0.551). Log NT-proBNP significantly decreased from baseline to 3 months and from baseline to 6 months of LVAD support (mixed effects joint test *p* = 0.005 unadjusted; *p* = 0.004 adjusted; Table 1, Table 2; Figure 1b) with the change occurring between baseline and 3 months (Figure 1). There was a significant within-participant association between log NT-proBNP and ALM, with a beta coefficient of −2.60 (95% confidence interval, CI, −3.75, −1.46; *p* < 0.001; Table 3). Within-participant decreases in log NT-proBNP values were associated with within-participant increases in ALM values. The relationship remained significant after adjustment for age, sex, and HF duration. The percent change in log NT-proBNP from baseline to 3 months was significantly negatively correlated with the percent change in ALM from baseline to 3 months: *r* = −0.60, 95% CI −0.82, −0.22, *p* = 0.003 (Figure 2a). At 6 months, the percent change in log NT-proBNP remained negatively correlated with the percent change in ALM, though the association was weaker and did not reach statistical significance: *r* = −0.45, 95% CI −0.79, 0.09, *p* = 0.092 (Figure 2d).

**Table 3:** Summary of Linear Mixed Effect Models for Relationship Between Appendicular Lean Mass and the Biomarker, Physical Activity, and Dietary Intake Parameters

Predictor variable	Adjusted*	Within-participant effect (95% CI)	*p*-value	Between-participant effect (95% CI)	*p*-value
Log NT-proBNP	No	−2.60 (−3.75, −1.46)	<0.001	0.51 (−5.45, 6.48)	0.862
Log NT-proBNP	Yes	−2.58 (−3.73, −1.44)	<0.001	−0.63 (−6.59, 5.34)	0.832
Log GDF-15	No	−0.97 (−2.07, 0.13)	0.083	−1.76 (−4.78, 1.26)	0.244
Log GDF-15	Yes	−0.96 (−2.06, 0.14)	0.086	−1.49 (−4.51, 1.53)	0.322
hsCRP	No	−0.02 (−0.03, 0.00)	0.018	0.00 (−0.07, 0.07)	0.981
hsCRP	Yes	−0.03 (−0.04, −0.02)	<0.001	−0.03 (−0.10, 0.04)	0.387
24 hours Steps^a^	No	0.11 (−0.06, 0.29)	0.195	0.05 (−0.65, 0.76)	0.876
24 hours Steps^a^	Yes	0.11 (−0.06, 0.29)	0.203	−0.06 (−0.70, 0.58)	0.852
24 hours Protein intake	No	−0.43 (−1.59, 0.73)	0.453	−4.02 (−7.94, −0.10)	0.045
24 hours Protein intake	Yes	−0.44 (−1.60, 0.72)	0.441	−5.27 (−8.53, −2.01)	0.002

* Adjusted for age, sex, and HF duration.

a Estimate is for 1,000 steps.

Log GDF-15 non-significantly decreased from baseline to 3 months and from baseline to 6 months of LVAD support (mixed effects joint test *p* = 0.250 unadjusted; *p* = 0.221 adjusted; Table 1, Table 2; Figure 1c). The point estimates for the within- and between-participant effects for the log GDF-15 model were both negative, suggesting participants with higher average GDF-15 values had a trend towards lower ALM values, although the threshold for significance was not reached (Table 3). However, the percent change in GDF-15 from baseline to 3 months was significantly associated with the percent change in ALM: *r* = −0.48, 95% CI −0.78, −0.02, *p* = 0.036 (Figure 2b). Similarly, at 6 months, the percent change in log GDF-15 remained significantly associated with the percent change in ALM, with a consistent negative correlation: *r* = −0.78, 95% CI −0.93, −0.40, *p* = 0.002 (Figure 2e).

hsCRP significantly decreased from baseline to 3 months and from baseline to 6 months of LVAD support (mixed effects joint test *p* < 0.001 unadjusted; *p* < 0.001 adjusted; Table 1, Table 2; Figure 1d) with the change occurring from baseline to 3 months. There was a significant within-participant association between hsCRP and ALM, with a beta coefficient of −0.02 (95% confidence interval, CI, −0.03, 0.00; *p* = 0.018; Table 3). Within-participant decreases in hsCRP values were associated with within-participant increases in ALM values. The relationship remained significant after adjustment for age, sex, and HF duration. However, the percent change in hsCRP from baseline to 3 months was not significantly associated with the percent change in ALM: *r* = −0.20, 95% CI −0.58, 0.25, *p* = 0.371 (Figure 2c). The percent change in hsCRP at 6 months was also unassociated with the percent change in ALM: *r* = −0.23, 95% CI −0.67, 0.34, *p* = 0.41 (Figure 2f).

### Habitual physical activity

There was a significant increase in average 24-hour steps from baseline to 3 months and from baseline to 6 months of LVAD support (Table 2 and Supplemental Table 2; Figure 1e). However, this increase in habitual physical activity was not significantly related to the increase in ALM (Table 3). The percent change in 24-hour steps from baseline to 3 months was not significantly associated with the percent change in ALM.

### Dietary intake

There was no significant increase in average 24-hour dietary protein intake from baseline to 3 or 6 months of LVAD support (Table 2 and Supplemental Table 2; Figure 1f) and no within-participant relationship to the increase in ALM (Table 3). The percent change in 24-hour protein intake from baseline to 3 months was not significantly associated with the percent change in ALM. The average total calorie, carbohydrate, and fat intakes also did not significantly change (Supplemental Table 2). There was a decrease in Short Nutritional Assessment Questionnaire scores (indicating a reduction in malnutrition risk) over the 6-month period of observation (Supplemental Table 2).

### Muscle strength, physical function, and patient-reported health status

Based on the mixed effects model joint test, there was a borderline non-significant increase in handgrip strength over the follow-up period (unadjusted *p* = 0.057, adjusted *p* = 0.064; Supplemental Table 2). There was a significant increase in each of the SPPB, 6MWT, and KCCQ summary scores, with most of these improvements occurring between the baseline and 3-month measurements (Supplemental Table 3). There was no statistically significant relationship between the increase in SPPB, 6MWT, or KCCQ and the increase in ALM (Supplemental Table 4).

## Discussion

In this prospective body composition study of patients with advanced HFrEF undergoing LVAD implantation, we observed several baseline associations between parameters representing candidate mechanisms for muscle wasting and DXA ALM, plus significant relationships between changes in some of these parameters and the increase in ALM during the first 6 months of LVAD support. There was a significant negative correlation between baseline ALM and NT-proBNP, followed by a decrease in NT-proBNP from baseline to 3 and 6 months of LVAD support that was associated with the increase in DXA ALM. Similar trends were seen for GDF-15, but the decrease over time and the inverse change relationship with ALM over the 6 months did not reach statistical significance. For hsCRP, there was a significant decrease from baseline to 3 and 6 months of LVAD support, and the decrease in hsCRP was associated with the increase in DXA ALM on mixed effects modeling, although on visual display of individual participant data in the scatterplot, this was likely driven by outliers. In contrast to these biomarkers, there were no statistically significant associations between changes in habitual physical activity or dietary intake and change in ALM over the same period. These findings support our hypothesis that muscle mass recovery correlates most closely with improved HF neurohumoral stability early after LVAD implantation, as represented by NT-proBNP recovery.

### HF neurohumoral stability and inflammation

In our previous study,^1^ we found that LVAD recipients with advanced HFrEF and a 52% baseline prevalence of muscle wasting had significant increases in FFM and ALM over the first 6 months of LVAD support. The main findings in our present study are that higher baseline NT-proBNP is associated with lower ALM and that a decrease in NT-proBNP over the first 6 months is strongly correlated with increased ALM, suggesting that recovery of lean mass is closely related to recovery from the neurohumoral activation state of advanced HFrEF***.*** It is well established that NT-proBNP is an excellent biomarker of HF severity and volume retention that has good prognostic utility for future HF hospitalizations and mortality events among patients with HFrEF.^18^ Our findings align with a prior investigation in which low axial skeletal muscle mass, as measured by magnetic resonance imaging, was independently associated with higher NT-proBNP among 205 outpatients with HFrEF or HF with preserved ejection fraction, with a coefficient of −0.305 (standard error 0.051, *p*-value 0.0001).^9^ NT-proBNP was more strongly related to muscle mass than adipose mass in this magnetic resonance imaging study.^9^ Such observations are corroborated by a registry analysis of outpatients with HF and NYHA II-III functional class in Japan, where a greater percent increase in BNP was associated with ≥5% weight loss over a 1 year period.^8^ The implication of these natriuretic peptide findings are that recovery of skeletal muscle in patients with HF appears to be highly dependent upon controlling neurohumoral activation and enhancing HF stability.

GDF-15 is a stress responsive cytokine that performs well as a prognostic HF biomarker and is more strongly associated with adverse outcomes in patients with HFrEF and chronic kidney disease than BNP.^19^, ^21^, ^31^ In addition, higher levels of GDF-15 have been associated with skeletal muscle wasting and lower physical performance in patients with cardiovascular conditions.^22^, ^32^, ^33^ Serum concentrations of both NT-proBNP and GDF-15 are known to significantly decrease after implantation of an LVAD, concurrent with improvements in other biomarkers of HF stability, but often do not normalize during LVAD support.^16^ Similarly, C-reactive protein and other inflammatory markers are elevated in advanced HFrEF and have been observed to improve after LVAD implantation^16^, ^34^; systemic inflammation is known to be a pathological contributor towards muscle wasting in chronic diseases.

Prior LVAD biomarker observations^14^, ^16^ found that patients who have greater early reductions in NT-proBNP after LVAD placement experience greater early weight recovery based on increases in BMI. The current study builds on this finding and demonstrates that recovery of DXA-imaged lean mass is inversely associated with NT-proBNP levels, which provides a more direct assessment of the skeletal muscle mass compartment than the BMI, which is influenced by fat and fluid compartments. The inverse relationship between NT-proBNP and ALM was the most consistent finding in this study: NT-proBNP and ALM values were inversely associated at baseline, NT-proBNP significantly decreased over the 6-month period while ALM significantly increased, and the decreases in log NT-proBNP values were significantly associated with increases in ALM values by both mixed effects modeling over 6 months as well as by percent change from baseline to 3 months of LVAD support. These findings cannot infer that the post-LVAD reductions in circulating NT-proBNP or hsCRP levels were the cause of the observed skeletal muscle recovery, and NT-proBNP is not currently known to have a direct effect upon skeletal muscle. However, there is limited data to suggest that HFrEF pharmacotherapies—which reduce the sympathetic-neurohumoral activation, for which NT-proBNP is a biomarker—can protect against unintentional weight loss.^35^, ^36^ A potential mechanistic link between greater neurohumoral activation and greater catabolism is therefore plausible and merits further evaluation.

Although GDF-15 levels in the current LVAD cohort showed trends towards a decrease during the first 6 months of support and an inverse relationship with 3-month ALM change, neither of the GDF-15 mixed effects models were statistically significant, potentially representing insufficient statistical power. There is, however, clinical relevance in these GDF-15 findings because a feasible future pharmacological intervention to promote muscle mass could be GDF-15 neutralization with antibodies such as ponsegromab, visugromab (CLT-002), or mAB2. Baseline ALM was negatively associated with GDF-15 levels in our study, suggesting that GDF-15 induced muscle degradation could be a contributor to wasting in advanced HFrEF. This therapeutic strategy is particularly interesting because chemotherapy-induced anorexia and weight loss is known to be attenuated in GDF-15 knockout mice and higher GDF-15 has been associated with lower extremity muscle endurance in another study of patients with HF and healthy adults.^23^, ^37^ Furthermore, a phase 2 study of the GDF-15 antibody ponsegromab recently achieved a dose-dependent weight gain for patients with cancer cachexia,^38^ raising the possibility of a therapeutic opportunity in patients with cardiac cachexia. Inflammation has long been a therapeutic target of interest in HFrEF, but to date interventions such as tumor necrosis factor alpha inhibition have shown lack of efficacy or even potential harm.^39^, ^40^

### Habitual physical activity

As anticipated, habitual physical activity, represented by 24-hour average steps, increased over the 6-month study period. However, this increase in habitual physical activity was not statistically associated with the increase in ALM; although the analysis could have been underpowered to detect an association, these results decrease the likelihood that gains in physical activity are a key contributor towards muscle mass recovery early after LVAD implantation. Regardless, weight-bearing and resistance activities are generally recognized as an important anabolic stimulus for maintenance or regain of skeletal muscle mass and physical functioning in patients with chronic diseases.^41^, ^42^ Therefore, cardiac rehabilitation and other exercise training opportunities remain an appropriate component of outpatient management for patients with HFrEF and LVAD recipients in specific.^43^ Of relevance to the current study, it has been reported that LVAD recipients with good functional status show enhanced muscle microvascular blood flow during exercise, per contrast-enhanced ultrasound, which was attributable to faster microvascular flux rate.^44^

### Dietary intake

Given that there was no significant increase in dietary protein intake, nor total calorie, carbohydrate, or fat intake, over the study period, we had no evidence that nutritional changes are responsible for the recovery of ALM in this cohort. It is currently uncertain whether inadequate dietary macronutrient intake contributes to skeletal muscle wasting in patients with HFrEF, and there are minimal published data on augmenting caloric or protein intake to prevent or reverse the wasting syndrome. Dietary protein intake has been estimated to be adequate for the majority of patients with HF based on the 0.8 g/kg/day adult intake recommendation from the Institute of Medicine.^45^ However, a small nitrogen balance study suggested patients with HF may require a higher protein intake of 1.1 g/kg/day,^46^ which was adopted as a recommendation by the American Academy of Nutrition and Dietetics.^47^ The dietary protein requirement for patients with HFrEF and catabolic weight loss has not been ascertained and could even be closer to the 1.5 g/kg/day intake recommended in the setting of critical illness.^48^, ^49^

By these standards, it is possible that the average protein intakes reported in this study at LVAD implantation, 3 months, and 6 months of LVAD support (0.98 ± 0.43, 1.08 ± 0.53, and 1.03 ± 0.69 g/kg/day, respectively) may be insufficient to meaningfully contribute towards skeletal muscle mass increases. Whether higher protein intake, such as 1.5 g/kg/day, early after LVAD implantation would accelerate muscle mass recovery is unknown. Three small oral protein or amino acid supplementation studies in patients with HF (sample sizes 29-38) suggest minor gains in edema-free weight or DXA FFM; one of these studies randomized patients with HFrEF to 1-alanyl-1-glutamine (8 g/day) plus polyunsaturated fatty acid (6.5 g/day) versus placebo for 3 months and reported increased FFM (54.4 ± 3.2 to 56.1 ± 2.5 kg, *p* = 0.04), although muscle function was unchanged.^50^, ^51^, ^52^ Although the current study did not detect any signal of dietary intake as a significant contributor towards ALM increases early after LVAD implantation, it remains possible that nutritional support may be an important component of a multi-modal approach towards muscle mass recovery, such as in combination with a pharmacological agent.

Limitations to this study include the small cohort size, the single-center design, and the predominance of male participants, which could collectively reduce the generalizability of these findings to the wider LVAD population. Differential follow-up periods were an inherent limitation within this study protocol, with significant missing data at the 3-month and 6-month visits predominantly due to cardiac transplantation during the first 6 months of LVAD support and participant preference. It remains possible that the observed increase in ALM and the relationship with NT-proBNP reduction was due to selection bias of the participants remaining in the study over time, although there were no deaths during the 6-month observation, and patients remaining on LVAD support without a transplantation option generally show greater clinical instability and frailty than those transplanted. Most importantly, the data presented is observational and cannot be interpreted to infer causal relationships or demonstrate mechanisms. Future clinical trials of pharmacological and nutritional interventions that may augment muscle mass recovery in patients with advanced HFrEF will be necessary for mechanistic insights.

In conclusion, this prospective observational study demonstrates that skeletal muscle recovery early during LVAD support is most closely associated with reductions in circulating NT-proBNP. Collectively the findings from this study indicate that the reversal of skeletal muscle wasting early after LVAD implantation is most closely related to improved HFrEF neurohumoral stability and inflammation, as opposed to increases in habitual physical activity or dietary intake.

## Disclosure statement

Amanda R. Vest, Joronia Chery, Alex Coston, Laura Telfer, Matthew Lawrence, Didjana Celkupa, Masashi Kawabori, Nathan LeBrasseur, and Edward Saltzman report no relevant relationships with industry. Michael S. Kiernan reports consultancy for Medtronic. Gregory Couper reports consultancy for Abbott and Medtronic. If there are other authors, they declare that they have no known competing financial interests or personal relationships that could have appeared to influence the work reported in this paper.

## Declaration of Competing Interest

The authors declare the following financial interests/personal relationships, which may be considered as potential competing interests: Amanda R. Vest reports financial support was provided by the National Institutes of Health. Amanda R. Vest reports financial support was provided by American Heart Association Inc.

## Financial support

Funding for this work was provided by an American Heart Association Scientist Development Grant (AHA grant 17SDG33660279). The pilot phase prior to the AHA study was funded by a Tufts Clinical and Translational Science Institute Pilot Grant (NIH Clinical and Translational Science award UL1TR002544). Study data were collected and managed using REDCap electronic data capture tools hosted at Tufts Clinical and Translational Science Institute, funded by NIH award UL1TR002544. Nathan LeBrasseur is funded by the National Institute of Aging (award R01 AG055529), outside the submitted work. Amanda R. Vest is currently funded by grants from the National Heart, Lung, and Blood Institute (award R01 HL167113) and from the National Center for Advancing Translational Sciences (award RC2 TR004377), outside the submitted work.

## Footnotes

Appendix A Supplementary data associated with this article can be found in the online version at doi:10.1016/j.jhlto.2025.100338.
