# Supplementary material for: Evaluation of potential mechanisms for skeletal muscle mass recovery early after left ventricular assist device implantation
Source: JHLT Open. 2025 Jul 4;9:100338. doi: 10.1016/j.jhlto.2025.100338 (PMC12340567; doi:10.1016/j.jhlto.2025.100338)
Supplement: Supplementary file 1 — Supplementary material [file mmc1.docx]

**SUPPLEMENTAL MATERIALS**

**Supplemental Table 1a: Baseline Characteristics of Cohort**

**Supplemental Table 1b: Medication Prescriptions at Study Visits**

**Supplemental Table 2: Physical Function, Activity, and Nutrition Over Study Visits**

**Supplemental Table 3. Summary of Linear Mixed Effect Models for Change Over Time in Muscle Strength and Patient Reported Health Status**

**Supplemental Table 4. Summary of Linear Mixed Effect Models for Relationship between Appendicular Lean Mass and Muscle Strength and Patient Reported Health Status Parameters**

**Supplemental Table 1a: Baseline Characteristics of Cohort**

| **Characteristic** | **All subjects (n = 30)** |
| --- | --- |
| Age, years | 56 ± 12 |
| Male sex | 28 (87%) |
| White race | 27 (90%) |
| Weight, kg (standing scales) | 80.8 ± 18.3 |
| Weight, kg (DXA) | 80.8 ± 19.4 |
| Body mass index, kg/m^2^ | 26.4 ± 5.4 |
| Weight change in 12 months pre- LVAD, % | -7.4 ± 8.9 |
| Ischemic HF etiology | 12 (40%) |
| Obesity (BMI ≥30 kg/m^2^) | 5 (17%) |
| Diabetes | 11 (37%) |
| Duration of HF, months * | 50 (13, 107) |
| Device brand   - HeartWare™ - HeartMate 3™ | 15 (50%)  15 (50%) |
| Transplant listed before or after LVAD implantation | 21 (70%) |
| Temporary mechanical support pre-LVAD | 15 (50%) |
| Intermacs class* | 2 (2, 3) |
| Albumin, g/dL | 3.4 ± 0.5 |
| Prealbumin, mg/dL | 19.6 ± 5.8 |
| Hemoglobin, g/dL | 10.1 ± 1.9 |
| Creatinine, mg/dL | 1.1 ± 0.3 |
| Estimated glomerular filtration rate (mL/min/1.73m^2^) | 80.1 ± 22.4 |
| Continuous variables expressed as means ± standard deviations, except for * values expressed as median (25^th^ percentile, 75^th^ percentile) due to non-normal distribution  Categorical variables expressed as number (percentage of cohort)  Abbreviations: BMI, body mass index; HF, heart failure; LVAD, left ventricular assist device | |
| Source: Reproduced from Vest AR, Wong WW, Chery J, Coston A, Telfer L, Lawrence M, Celkupa D, Kiernan MS, Couper G, Kawabori M and Saltzman E. Skeletal Muscle Mass Recovery Early After Left Ventricular Assist Device Implantation in Patients With Advanced Systolic Heart Failure. *Circ Heart Fail*. 2022:101161CIRCHEARTFAILURE121009012. doi:10.1161/CIRCHEARTFAILURE.121.009012. | |

**Supplemental Table 1b: Medication Prescriptions at Study Visits**

| **Medication** | **Baseline (n = 30)** | **3 Months (n = 28)** | **6 Months (n = 20)** |
| --- | --- | --- | --- |
| Standing Loop Diuretics  (Furosemide, Torsemide, Bumetanide) | 21 (70%) | 18 (64%) | 12 (60%) |
| Beta Blockers  (Metoprolol XL, Carvedilol, Bisoprolol) | 6 (20%) | 10 (36%) | 7 (35%) |
| ACE/ARB/ANRI  (Lisinopril, Enalapril, Captopril, Losartan, Valsartan, Candesartan, Sacubitril-Valsartan) | 9 (30%) | 7 (25%) | 7 (35%) |

**Supplemental Table 2: Physical Function, Activity, and Nutrition Over Study Visits**

| **Physical Function and Activity** | **Baseline**  **(n = 30)** | **3 Months**  **(n = 27)**  ***Mean Change from Baseline (95% CI)*** | **6 Months**  **(n = 20)**  ***Mean Change from Baseline (95% CI)*** |
| --- | --- | --- | --- |
| Kansas City Cardiomyopathy Questionnaire summary score | 44 ± 18 | 62 ± 22  *19.33 (11.56, 27.10)* | 62 ± 20  *19.09 (9.55, 28.63)* |
| Handgrip strength, kg | 27 ± 8.9 | 28 ± 9.5  *2.00 (-0.78, 4.78)* | 29 ±10.3  *3.53 (-0.08, 7.13)* |
| 4-meter walk, seconds | 5.8 ± 2.6 | 4.2 ± 1.2  *-1.47 (-2.56, -0.38)* | 4.3 ± 1.2  *-1.07 (-2.15, 0.01)* |
| Short Physical Performance Battery score | 7 ± 3 | 10 ± 2  *3 (2, 4)* | 10 ± 2  *3 (2, 4)* |
| 6-Minute walk test distance, meters | 178 ± 106 | 315 ± 103  *130 (84, 175)* | 332 ± 120  *143 (79, 206)* |
| 24-hour average steps | 6545 ± 2268 | 8646 ± 3208  *2398 (1134, 3662)* | 9830 ± 4437  *3668 (1402, 5935)* |
| Average percent active time, % | 43.5 ± 10.1 | 47.0 ± 11.4  *5.29 (-0.71, 11.28)* | 48.9 ± 13.2  *6.37 (-2.60, 15.3)* |
| **Nutrition** | **Baseline**  **(n = 27)** | **3 Months**  **(n = 27)**  ***Mean Change from Baseline (95% CI)*** | **6 Months**  **(n = 19)**  ***Mean Change from Baseline (95% CI)*** |
| 24-hour average calorie intake, kcal | 1778 ± 616 | 1842 ± 739  *40 (-264, 344)* | 1717 ± 718  *-162 (-551, 227)* |
| 24-hour average calorie intake, kcal per kg bodyweight | 23.51 ± 9.77 | 23.97 ± 11.04  *0.71 (-3.52, 4.94)* | 20.37 ± 9.98  *-2.65 (-7.98, 2.69)* |
| 24-hour average protein intake, gram per kg bodyweight | 0.98 ± 0.43 | 1.08 ± 0.53  *0.10 (-0.10, 0.31)* | 1.03 ± 0.69  *0.08 (-0.24, 0.39)* |
| 24-hour average carbohydrate intake, grams (and as a % of 24- hour average caloric intake) | 230.1± 87.9  51.37% | 215± 98.8  *-15.48 (-58.59, 27.64)*  46.7% | 196.9 ± 87.1  *-37.71 (-104.5, 29.10)*  47.0% |
| 24-hour average fat intake, % of 24- hour average caloric intake | 64.92± 24.18  33.0% | 73.23± 34.20  *6.52 (-7.89, 20.93)*  35.27% | 66.88 ± 34.49  *-0.75 (-23.58, 22.09)*  34.19% |
| Short Nutritional Assessment Questionnaire score | 4 ± 2 | 3 ± 2  *-0.6 (-1.6, 0.4)* | 2 ± 2  *-2.2 (-3.6, -0.7)* |
| Abbreviations: CI, confidence interval; CRP, C-reactive protein  Continuous variables expressed as means ± standard deviations  Changes from baseline expressed as mean change and (95% confidence interval) | | | |
| Source: In part, reproduced from Vest AR, Wong WW, Chery J, Coston A, Telfer L, Lawrence M, Celkupa D, Kiernan MS, Couper G, Kawabori M and Saltzman E. Skeletal Muscle Mass Recovery Early After Left Ventricular Assist Device Implantation in Patients With Advanced Systolic Heart Failure. *Circ Heart Fail*. 2022:101161CIRCHEARTFAILURE121009012. doi:10.1161/CIRCHEARTFAILURE.121.009012. | | | |

**Supplemental Table 3. Summary of Linear Mixed Effect Models for Change Over Time in Muscle Strength and Patient Reported Health Status**

| **Variable** | **Adjusted*** | | **3-month change (95% CI)** | | **P-value** | **6-month change (95% CI)** | | **P-value** | **P-value joint test** | |  |
| --- | --- | --- | --- | --- | --- | --- | --- | --- | --- | --- | --- |
| Handgrip, kg | No | | 1.85 (-0.68, 4.38) | | 0.147 | 3.45 (0.60, 6.30) | | 0.019 | 0.057 | |  |
| Handgrip, kg | Yes | | 1.91 (-0.61, 4.44) | | 0.134 | 3.33 (0.49, 6.18) | | 0.023 | 0.064 | |  |
| SPPB | No | | 3.10 (2.14, 4.05) | | <0.001 | 3.18 (2.13, 4.23) | | <0.001 | <0.001 | |  |
| SPPB | Yes | | 3.16 (2.20, 4.12) | | <0.001 | 3.20 (2.14, 4.26) | | <0.001 | <0.001 | |  |
| 6MWT, meters | No | | 138.03 (95.53, 180.52) | | <0.001 | 149.02 (101.16, 196.89) | | <0.001 | <0.001 | |  |
| 6MWT, meters | Yes | | 139.21 (96.36, 182.05) | | <0.001 | 148.64 (100.44, 196.84) | | <0.001 | <0.001 | |  |
| KCCQ | No | | 20.47 (13.47, 27.46) | | <0.001 | 20.78 (12.99, 28.58) | | <0.001 | <0.001 | |  |
| KCCQ | Yes | | 20.54 (13.53, 27.56) | | <0.001 | 20.68 (12.87, 28.50) | | <0.001 | <0.001 | |  |
| * For age, sex, and HF duration | |  | |  | | |  | | |  | |

Abbreviations: 6MWT, 6-minute walk test; CI, confidence interval; KCCQ, Kansas City Cardiomyopathy Questionnaire; kg, kilograms; SPPB, short physical performance battery

**Supplemental Table 4. Summary of Linear Mixed Effect Models for Relationship between Appendicular Lean Mass and Muscle Strength and Patient Reported Health Status Parameters**

| **Variable** | **Adjusted*** | **Within-participant effect (95% CI)** | **P-value** | **Between-participant effect (95% CI)** | | **P-value** |  |
| --- | --- | --- | --- | --- | --- | --- | --- |
| SPPB | No | 0.10 (-0.17, 0.38) | 0.439 | -0.07 (-0.97, 0.84) | | 0.884 |  |
| SPPB | Yes | 0.09 (-0.18, 0.36) | 0.509 | -0.17 (-0.99, 0.66) | | 0.684 |  |
| 6MWT, meters | No | 0.003 (-0.003, 0.009) | 0.354 | -0.003 (-0.021, 0.014) | | 0.718 |  |
| 6MWT, meters | Yes | 0.003 (-0.004, 0.009) | 0.411 | -0.005 (-0.021, 0.011) | | 0.530 |  |
| KCCQ | No | -0.004 (-0.04, 0.03) | 0.813 | -0.06 (-0.16, 0.05) | | 0.269 |  |
| KCCQ | Yes | -0.006 (-0.04, 0.03) | 0.720 | -0.08 (-0.17, 0.01) | | 0.069 |  |
| * For age, sex, and HF duration  ^1^ Estimate is for 1,000 steps  # Relationship between handgrip strength and ALM not tested, due to non-significance of handgrip strength change over time | | |  |  |  | | |

Abbreviations: 6MWT, 6-minute walk test; CI, confidence interval; KCCQ, Kansas City Cardiomyopathy Questionnaire; SPPB, short physical performance battery
